# Controlling Cell Migratory Patterns Under an Electric Field Regulated by a Neural Network-Based Feedback Controller

**DOI:** 10.3390/bioengineering12070678

**Published:** 2025-06-20

**Authors:** Giovanny Marquez, Mohammad Jafari, Manasa Kesapragada, Kan Zhu, Prabhat Baniya, Yao-Hui Sun, Hao-Chieh Hsieh, Cristian O. Hernandez, Mircea Teodorescu, Marco Rolandi, Min Zhao, Marcella Gomez

**Affiliations:** 1 Department of Applied Mathematics, Baskin School of Engineering, University of California Santa Cruz, Santa Cruz, CA 95064, USA; 2 Department of Earth and Space Sciences, Columbus State University, Columbus, GA 31907, USA; 3 Department of Dermatology, University of California Davis, Sacramento, CA 95816, USA; 4 Department of Ophthalmology & Vision Science, University of California Davis, Sacramento, CA 95817, USA; 5 Department of Electrical and Computer Engineering, Baskin School of Engineering, University of California Santa Cruz, Santa Cruz, CA 95064, USA

**Keywords:** feedback control, galvanotaxis, predictive biology, wound healing

## Abstract

Electric fields (EFs) are widely employed to promote tissue regeneration and accelerate wound healing. Despite extensive study, the cellular responses elicited by EFs are complex and not well understood. The present work focuses on cell migration—a process essential to organismal development, immune surveillance, and repair—and seeks to achieve its precise, closed-loop regulation. Effective control is impeded by (i) the nonlinear and stochastic nature of migratory dynamics and (ii) safety constraints that restrict the admissible EF magnitude. To address these challenges, we reformulate a neural network (NN) feedback controller previously developed for single-cell membrane-potential regulation and adapt it to guide population-level cell migration. A projection operator is embedded into the NN weight-update law to prevent maladaptive learning that arises when the control signal saturates at its EF limit. Numerical simulations confirm that the modified controller maintains accurate trajectory tracking under saturation and outperforms the original NN design. Finally, we demonstrate a proof-of-concept by implementing the controller in vitro to direct the electrotactic migration of naïve macrophages in 2D culture under a unidirectional EF. For the in vitro experiments, we compare performance to the standard proportional–integral–derivative (PID) controller.

## 1. Introduction

Regulating cell migration is critical to many biological processes, including development, tissue homeostasis, and disease progression [1]. Impaired migration contributes to pathologies such as cancer and autoimmune disorders [1]. Recent advances indicate that cell movement can be modulated externally through feedback control systems that integrate biological sensors with actuators [2,3]. Bioelectronic devices, which enable real-time modulation of cellular activity, have already shown considerable promise in applications ranging from blood-glucose regulation and stem-cell manipulation to electrical stimulation and targeted drug delivery [4,5,6,7].

Electric fields (EFs) constitute a principal guidance cue for cell migration through galvanotaxis, a mechanism that is especially important during wound healing [8,9]. To exploit this response, “smart” bandages have been engineered to augment the endogenous EF with an externally applied stimulus, thereby accelerating tissue repair [10,11]. Although numerous in vitro studies have characterized how EFs influence migratory behavior [8,12,13], relatively few have addressed active control of that behavior. One notable exception is a platform that steered keratinocyte migration in two-dimensional culture using predefined EF waveforms [14]. Because EFs can also induce undesirable phenotypic changes [15,16], precise regulation is critical to maximize therapeutic benefit while minimizing off-target effects. Closed-loop feedback control offers a compelling strategy to modulate EFs dynamically and achieve targeted migratory outcomes.

**Figure 1:**
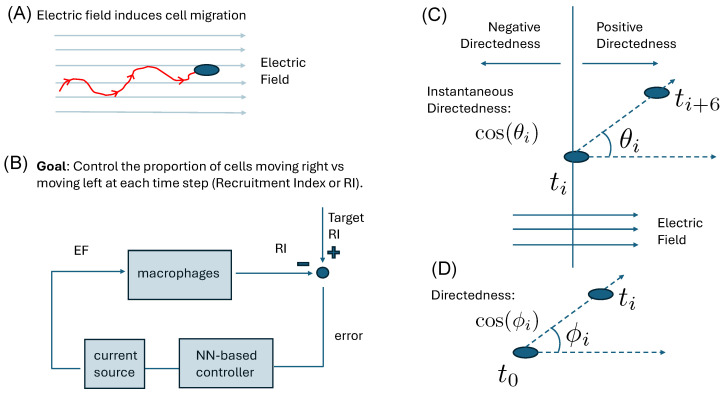
(**A**) Cell migration is induced by the application of an electric field in a process known as galvanotaxis. (**B**) Feedback control is implemented to automate the regulation of the applied electric field to achieve a desired collective behavior. A neural network controller adjusts the EF strength by way of a current source based on the measured error between the observed response and target behavior of a population of cells. (**C**) Cell behavior is quantified by computing the instantaneous directedness of each cell at each time step. This, in turn, is used to compute the Recruitment Index (RI), which reflects the proportion of cells moving right vs. left. (**D**) A commonly used definition of directedness considers cell displacement with respect to its original position, which can be used when there is no polarity switch.

Effective feedback control of electrotactic migration requires coordinated sensing and actuation. In our framework, the actuator is the externally applied EF, whose magnitude is modulated in real time by the controller using data from an imaging sensor (Figure 1). Single-cell microscopy supplies continuous positional information to close the loop. A naïve strategy would apply the maximum permissible EF to accelerate migration, but excessive stimulation risks off-target effects. The objective, therefore, is to deliver the smallest EF that reliably drives the desired motility while limiting exposure.

Implementing feedback in living systems is challenging because their dynamics are nonlinear, stochastic, and time-varying [17,18]. Controllers must also remain robust to environmental disturbances and to drift in sensor or actuator performance across experiments. Neural network (NN) controllers can address these issues by adapting online without an explicit mechanistic model [19]. However, NNs are vulnerable to control-signal saturation: when the required EF exceeds safety limits, the output must be clipped, and the resulting weight updates can degrade performance. Traditional anti-windup and sliding-mode strategies partly mitigate saturation [20], but they struggle under the long sampling intervals and delays typical of biological assays.

We propose a neural network (NN) feedback controller expressly engineered to operate under control-signal saturation. A projection operator embedded in the NN weight-update law constrains the commanded EF within the device’s safe operating range, thereby preventing maladaptive learning when saturation occurs. Controller efficacy is first quantified in silico using a stochastic model of electrotactic cell migration. We then demonstrate the practical applicability of our approach in vitro, where we successfully control macrophage migration through EF regulation, utilizing time-lapse microscopy in combination with image processing and cell tracking software.

## 2. Materials and Methods

In this section, we present in detail the quantitative methods used to analyze data, the experimental methods, and relevant background on the previously used NN controller.

### 2.1. Image Analysis Tool for Image Processing/Cell Tracking

**Figure 2:**
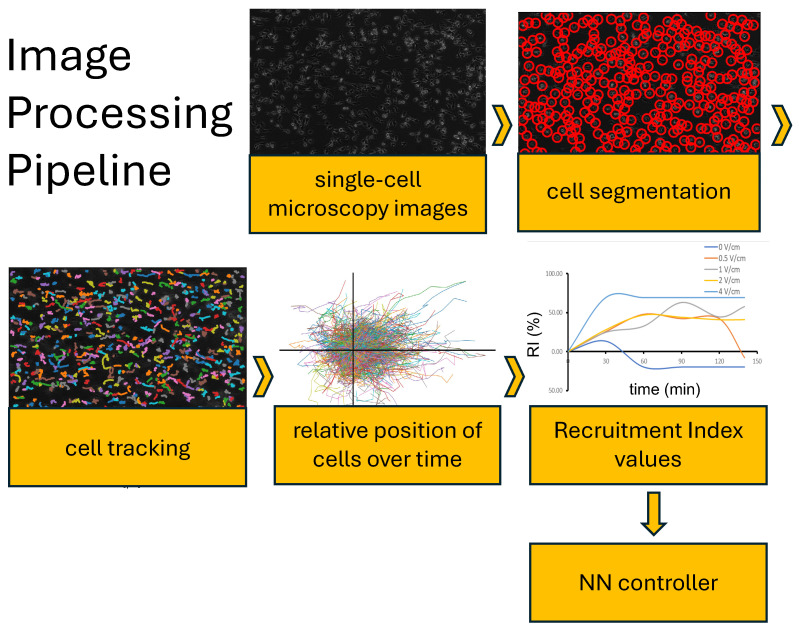
Depiction of the schematic of the stages of the Image Analysis Tool used in this study. Cell tracking is achieved through image processing to identify each unique cell and track its position over time. From the trajectory of the cell, additional metrics can be computed.

To analyze single-cell microscopy data, we develop an image analysis tool (refer to Figure 2) that consists of a script written in Python 3 designed to track the cell position over time and compute metrics of interest. The inputs for the image analysis tool consist of images taken using a microscope every 5 min. Then Trackpy [21], which is a particle tracking library in Python that operates for two and higher dimensions, is used for detecting and monitoring the cells within these images over time. Next, the trajectories of the cells are extracted and examined in order to calculate the metrics described in the next section. The calculated metrics are then stored in a comma-separated values (CSV) file format. The CSV file is then accessed by the NN-based algorithm for real-time feedback control.

### 2.2. Quantification of Cellular Response

First, the speed of the cell is approximated in order to filter out stationary cells or cells with low migration speed (i.e., less than the 25th percentile). The speed at which the cells migrate is determined by the ratio of the total distance traveled to the elapsed time, calculated every five minutes as follows:

(1) $$\mathrm{Speed}\;\mathrm{of}\;\mathrm{Migration}=\frac{\sum_{i}^{n}\sqrt{{\hat{x}}_{i}^{2}+{\hat{y}}_{i}^{2}}}{i\times 5}$$

where ${\hat{x}}_{i}$ and ${\hat{y}}_{i}$ are the relative positions of cells at each time step.

Next, we compute the directedness of cell movement. Directedness is defined as the cosine of the angle that the cell trajectory makes with the direction of the EF (see Figure 1C,D). If the cell trajectory is perfectly aligned with the EF, then the value is 1, and if it is perfectly aligned but in the opposite direction, the value is −1. The instantaneous directedness of each cell’s motion is computed every 30 min, which corresponds to analyzing 6 frames ahead, as shown below (see Figure 1C):

(2) $$cos\theta_{i}=\frac{{\hat{x}}_{i+6}-{\hat{x}}_{i}}{\sqrt{{\left({\hat{x}}_{i+6}-{\hat{x}}_{i}\right)}^{2}+{\left({\hat{y}}_{i+6}-{\hat{y}}_{i}\right)}^{2}}},$$

where ${\hat{x}}_{i}$ and ${\hat{y}}_{i}$ are the relative position values of a cell at time ${\hat{t}}_{i}$.

The third metric we compute is the Recruitment Index (RI), which quantifies the percentage of cells moving towards the anode vs. the cathode for different EF strengths. If all cells migrate toward the anode, the RI will be 100%, while it will be −100% if all cells move toward the cathode. The Recruitment Index (RI) is determined by the following formula:

(3) $$\mathrm{RI}=\frac{C_{A}-C_{C}}{C_{T}}\times 100$$

where

- $C_{A}$ are those cells with directedness >0.01.
- $C_{C}$ are those cells with directedness <−0.01.
- $C_{T}$ is the total cell count (including those with directedness between −0.01 and 0.01).

This metric is used in the NN-based feedback algorithm for tracking purposes.

### 2.3. Preparing Macrophages

**Figure 3:**
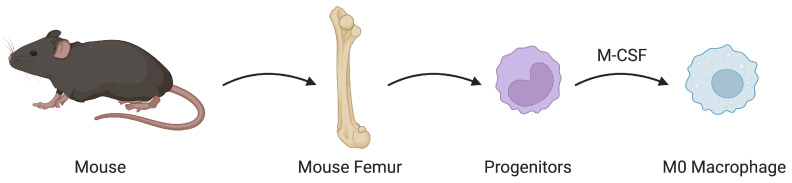
Schematic depicting how macrophages were prepared. Bone marrow cells are extracted from the tibia or femur of mice. These cells are then differentiated into naive macrophages.

For this study, we consider the application to naive macrophages. In accordance with the established protocols detailed by Sun et al. [22], the bone marrow-derived macrophages (BMDMs) were produced and isolated in vitro (refer to Figure 3). All procedures were approved by the Institutional Animal Care and Use Committee of UC Davis (protocol #23542). In brief, bone marrow cells were extracted from the tibia or femur of C57BL/6 mice and cultured in Dulbecco’s Modified Eagle Medium (DMEM, Invitrogen, Carlsbad, CA, USA) enriched with 10% Fetal Bovine Serum (FBS, Invitrogen) and a 1× Antibiotic-Antimycotic solution (Invitrogen). The culture medium was augmented with 20% l-929 conditioned medium containing M-CSF for 6 days, followed by an additional 24 h incubation without the conditioned medium. Adherent macrophages were gathered by careful scraping with a cell scraper and were then plated in electrotaxis chambers for subsequent experimentation. The viability of the cells was evaluated using trypan blue staining. C57BL/6 mice were sourced from Jackson Laboratories and were housed in a specific pathogen-free environment at the University of California, Davis (UCD), adhering to a strict 12 h light cycle and being fed a standard chow diet.

### 2.4. Experimental Setup

**Figure 4:**
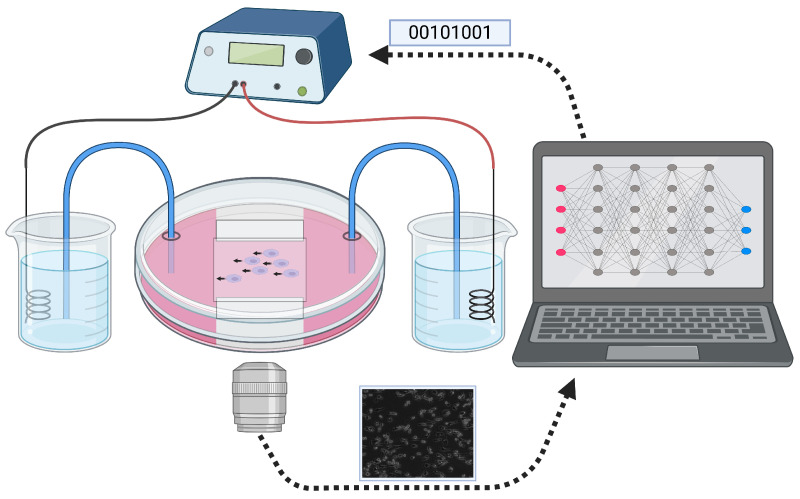
Depiction of the schematic of the experimental setup used in this study. A current source is connected to an electrotaxis chamber housing cell cultures and is set up to apply an EF of up to 4 V/cm. Microscopy images are acquired and processed in real time, and the corresponding computed metrics are fed into the NN controller, which automatically adjusts the applied current within specified bounds.

The schematic of the experimental setup utilized in this study is illustrated in Figure 4. Macrophages were cultured in an electrotaxis chamber based on tissue culture dishes (Corning) to achieve optimal in vitro migration results. As described in previous research [23], the chamber was constructed using glass strips and sealed with high vacuum grease. A DC (i.e., Direct Current) electric field (EF) of up to 4 V/cm across the chamber was applied via Ag/AgCl electrodes to stimulate electrotactic movement of the cells. The actual voltage drop across the chamber was confirmed before and after each experiment.

The power supply system comprises a Keithley current source. This current source directs current through Agar bridges and Steinberg’s solution. This setup results in a uniform electric field being applied to the macrophages within the electric field chamber. The images of the cells were captured every 5 min using a microscope. Finally, the images were then saved on a computer.

This setup was used to monitor the response of cells to a constant electric field and also used in the closed-loop control experiments with the proposed control algorithm to automatically adjust the applied current and, hence, EF.

In the closed-loop experiments, a MATLAB 2019b script, which analyzes the cellular response in real time (see Section 2.2), was run on the same computer; the script feeds this information into the NN-based control algorithm, and computes the next current value to apply to achieve the desired cellular response. The current value is transmitted to the current source via a serial cable linked to the computer.

### 2.5. Neural Network Controller Overview

Previous studies have demonstrated that adaptive neural network (NN) controllers updated in real time can effectively regulate biological systems even when no mechanistic model is available and system conditions vary [19,24]. Jafari et al. [19] specifically introduced an adaptive feedback controller based on a radial basis function neural network (RBF-NN) to modulate cellular responses on-the-fly. An RBF-NN is a three-layer, feed-forward architecture comprising an input layer, a hidden layer of radial basis units, and a linear output layer. Each hidden-layer neuron employs a Gaussian activation function, providing localized feature representation and facilitating universal function approximation. The Gaussian function takes on the following form:

(4) $$\Phi (||z\left(t\right)-c_{i}\left||\right)=e^{\frac{-∥z\left(t\right)-c_{i}{∥}^{2}}{\beta_{i}^{2}}}$$

The *i*th neuron consists of the input vector z(t), variance $\beta_{i}$, and the center vector $c_{i}$. The computed output of the RBF-NN is

(5) $$\Omega \left(z\left(t\right)\right)=\sum_{i=1}^{M}W_{i}\left(t\right)\Phi (||z\left(t\right)-c_{i}||),$$

where *M* represents the number of neurons in the hidden layer and $W_{i}$ denotes the corresponding weights.

This neural network’s objective is to learn a feedback control law *u* for the given system:

(6) $$\begin{array}{c} \left\{\begin{array}{c} \dot{x}=f\left(x,u\right)+\delta \left(t\right)\\ y=x. \end{array}\right. \end{array}$$

The goal is to ensure that the measured output *y* tracks a specified reference value *r*. Specifically, the aim is to reduce the error e(t)=r(t)−y(t), where *e* represents the discrepancy between the reference *r* and the output *y*. The function *f* is defined over a fixed domain where $u_{min}<u<u_{max}$ and is characterized as a continuously differentiable and strictly monotonic function in terms of *u*, with the properties f(x,0)=0 and $f_{min}<f\left(x,u\right)<f_{max}$, where $f_{min}<0$ and $f_{max}>0$.

To adjust the parameters of the RBF, the gradient of the cost function $C\left(t\right)=\frac{1}{2}e{\left(t\right)}^{2}$ is utilized. By calculating $\frac{\partial C}{\partial W}$, the weights $W_{i}$ are modified following the update law mentioned in the following [19]:

(7) $${\dot{W}}_{i}\left(t\right)=e\left(t\right)\Phi (||z\left(t\right)-c_{i}||),$$

where e(t) is the system error to be minimized. We have included the Lyapunov stability analysis in Appendix A.

For implementation purposes, we let

(8) $${\dot{W}}_{i}\approx \frac{W_{i}\left(t+T_{s}\right)-W_{i}\left(t\right)}{\gamma },$$

where γ is the learning rate of the weights and $T_{s}$ is the sampling time. Using Equations (7) and (8) gives the following discrete-time update law:

(9) $$W_{i}\left(t+T_{s}\right)=W_{i}\left(t\right)+\gamma e\left(t\right)\Phi (||z\left(t\right)-c_{i}||).$$

The update law in Equation (7) is susceptible to control-signal saturation: when the commanded input exceeds actuator limits, it is clipped, the tracking error cannot reach zero, and the weights of the neural network continue to diverge, degrading performance. Avoiding these effects typically demands meticulous tuning of initial weights and learning rates. Building on prior work, we therefore integrate a projection operator into the update law, constraining parameter growth and averting maladaptive learning whenever the system dwells at saturation for extended intervals.

## 3. Results

In this section, we derive and apply the NN controller, updated with a revised law, to our in silico platform introduced in Section 3.1. We also demonstrate an in vitro implementation as a proof-of-concept to validate the practical applicability of the controller to control the migration of naïve macrophages.

### 3.1. Qualitative Stochastic Model of Cell Directedness

In order to assess the performance of our control law in silico, we introduce and utilize a qualitative stochastic model describing cell motility in response to an electric field. The state modeled here is cell directedness, from which we can calculate the recruitment index (RI). The relationship between cell directedness and EF is already established for various cell types [13].

**Figure 5:**
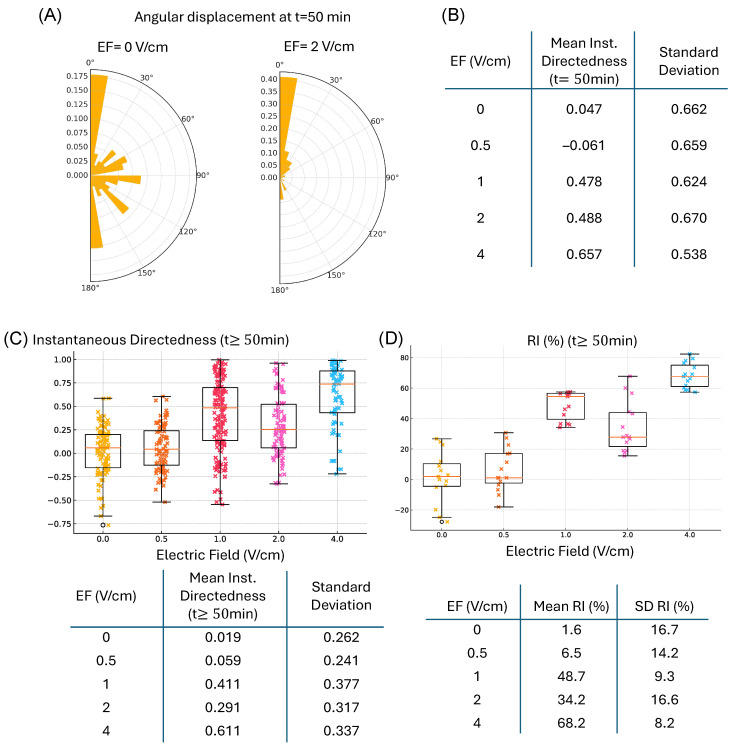
(**A**) A snapshot of the angular displacement of cells at time t = 50 min for two different electric field strengths. (**B**) A table with the computed mean instantaneous directedness and corresponding standard deviation across cells for each EF strength at t = 50 min. (**C**) The distribution of the instantaneous directedness of all cells for all times greater than or equal to 50 min after steady state dynamics are achieved. (**D**) The distribution of the recruitment index for all times greater than or equal to 50 min after steady state dynamics are achieved.

The in vitro data used to inform the model is generated using the same experimental setup described in the methods section, except with constant EF applied instead of having the feedback control algorithm dynamically change the EF. Figure 5 shows the computed instantaneous directedness (Figure 1C) and recruitment index (RI) for all tracked cells in experiments for constant values of EF. We have one experimental run for each EF value. We tracked the following number of cells for 120 min in each experiment (24 images): 101 cells at 0 V/cm, 89 cells at 0.5 V/cm, 167 cells at 1 V/cm, 90 cells at 2 V/cm, and 68 cells at 4 V/cm. Preliminary analysis revealed substantial variability in the directedness metric, whereas the recruitment index (RI) shows a markedly tighter correlation with EF magnitude. Consequently, our feedback loop targets RI directly.

**Figure 6:**
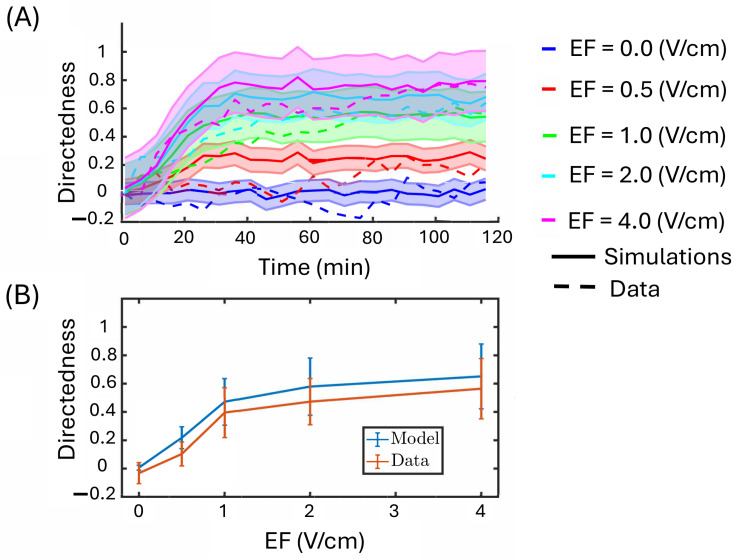
Model simulations are compared to experimental data. Modeling results consist of the same number of simulations as those for macrophage data (i.e., 101 cells at 0 V/cm, 87 cells at 0.5 V/cm, 167 cells at 1 V/cm, 90 cells at 2 V/cm, and 68 cells at 4 V/cm). In the data, we consider all cells tracked for 24 frames (i.e., 101 cells at 0 V/cm, 87 cells at 0.5 V/cm, 167 cells at 1 V/cm, 90 cells at 2 V/cm, and 68 cells at 4 V/cm). (**A**) The directedness values are plotted across time for comparison. The solid line is the mean directedness for all simulated cells, and the shaded region represents the 95% confidence interval. The dashed line is the mean directedness computed at each time step in the data. (**B**) The mean directedness and corresponding standard deviation are computed for each EF.

To fit the model, we consider the temporal trajectories of the cell migration patterns. Figure 6 shows the computed directedness of naive macrophages over time under a constant EF from single-cell microscopy data. Note that this is not the instantaneous directedness but rather the traditional metric used, illustrated in Figure 1D. This metric is less noisy and easier to use for data fitting purposes.

The model used for in silico studies is the following:

(10) $$d\left(t\right)=\frac{1}{1+e^{-(t-\tau )}}\times \frac{\nu }{s}\times \left[\left(2\times rand-1\right)+\frac{\frac{\mathrm{EF}}{s}+\mathrm{sign}\left(\mathrm{EF}\right)\times s\times {\left|\mathrm{EF}\right|}^{p}}{{\left|\mathrm{EF}\right|}^{p}+1}\right]$$

(11) $$p=\left\{\begin{array}{c} +1,\;\mathrm{if}\;\mathrm{EF}=\;\;0\\ \frac{1}{\left|\mathrm{EF}\right|},\;otherwise \end{array}\right.$$

(12) $$d\left(t\right)=\left\{\begin{array}{c} +1,\;\mathrm{if}\;d(t)>\;\;1\\ -1,\;\mathrm{if}\;d(t)<-1\\ d(t),\;otherwise \end{array}\right.$$

Here, d(t) is the directedness as a function of time. Let ν∈{−1,1} represent the direction in which the cell migrates under the influence of an electric field, specifically whether it moves towards or against the anode. The first term in Equation (10) captures the random motion of the cell, while the second term captures a bias induced by an electric field. The strength of the electric field applied to the cell is denoted as EF. The variable *s* indicates the strength of the cell’s movement towards the cathode under an electric field. A value of two implies that the cell’s directedness is not biased by an electric field.

The random function generator rand selects values from 0 to 1 from a uniform distribution and introduces stochasticity into the cell’s movement. A factor of 2 scales the random value so that it spans the range [−1, 1], representing all possible directions, with no bias toward one direction. The parameter *p* is defined as indicated in Equation (11) and acts to saturate the influence of an increasing EF strength. Additionally, τ is a delay parameter incorporated into the model to account for the initial delay in cellular response. Cells require time to respond when they are first exposed to an electric field (EF). The parameter *s* is set to three, which makes it easier for the cells to move toward the cathode with small electric field (EF) strengths. The delay parameter τ is set to four to align with the transient behavior observed in the naïve macrophage data. As directedness values should range between −1 and 1, Equation (12) ensures that any derived values are constrained within these limits by setting them to the minimum or maximum when necessary. Figure 6 shows the comparison between the model simulations and experimental data.

The number of cells utilized to fit the mathematical model across various electric field (EF) strengths is as follows: 101 cells at 0 V/cm, 87 cells at 0.5 V/cm, 167 cells at 1 V/cm, 90 cells at 2 V/cm, and 68 cells at 4 V/cm. We only considered cells tracked for all 24 frames. In Figure 6, plot (A) shows the mean directedness computed across all cells as a function of time (simulations and data). Plot (B) shows the mean directedness value and the corresponding standard deviation for each electric field (EF) strength.

### 3.2. Adapted Neural Network Controller

To address saturation in control systems, we introduce a projection operator into the neural network’s weight updates. This approach helps mitigate the effects of saturation and ensures that control inputs remain within the operational limits of the devices. To avoid having the controller output stray too far from acceptable values, the control update law is modified when the control output exceeds permissible limits. If the control output goes beyond the upper limit (UL) or falls below the lower limit (LL), a projection method is utilized to down-regulate the updates of the neural network weights. The improved updated law is as follows:

(13) $$\begin{array}{c} {\dot{W}}_{i}=\left\{\begin{array}{c} \gamma_{a}\Phi (||z-c_{i}||)e\\ -\mathrm{sign}\left(e\right)\alpha_{a}\frac{W_{i}W_{i}^{T}\Phi (||z-c_{i}\left||\right)}{∥W_{i}∥}e,\;\;\mathrm{if}\;u>UL\\ \gamma_{b}\Phi (||z-c_{i}||)e\\ +\mathrm{sign}\left(e\right)\alpha_{b}\frac{W_{i}W_{i}^{T}\Phi (||z-c_{i}\left||\right)}{∥W_{i}∥}e,\;\;\mathrm{if}\;u<LL\\ \;\gamma \Phi (||z-c_{i}||)e,\;\;\mathrm{otherwise}. \end{array}\right. \end{array}$$

This prevents the control signal from deviating excessively from the limits set by the experimental setup. By doing so, the control value can respond promptly when a change is needed.

Assigning appropriate values to the parameters $\gamma ,\gamma_{a}\gamma_{b},\alpha_{a},$ and $\alpha_{b}$ will prevent the weights from growing too quickly or push the system back within the specified control bounds. We can see this is the case by simplifying Equation (13), which reduces to

(14) $$\begin{array}{c} {\dot{W}}_{i}=\left\{\begin{array}{c} \Phi (||z-c_{i}||)e(\gamma_{a}-\mathrm{sign}\left(e\right)\alpha_{a}∥W∥)\;\mathrm{if}\;u>UL\\ \Phi (||z-c_{i}||)e(\gamma_{b}+\mathrm{sign}\left(e\right)\alpha_{b}∥W∥)\;\mathrm{if}\;u<LL\\ \;\gamma \Phi (||z-c_{i}||)e,\;\;\mathrm{otherwise}. \end{array}\right. \end{array}$$

From Equation (14), we see that values for $\alpha_{a,b}∥W∥$ and/or $\gamma_{a,b}$ are what dictate the growth of the weights along with the sign of the error. The projection operator acts like a feedback control law on the weights to prevent them from moving away from a set value when the controller output exceeds specified bounds. We note the direct relation between the sign of the weights and the induced direction of motion of cells. This relationship is due to the controller output being a linear combination of the weights and the Gaussian function (Φ). The Gaussian function (Φ) will always output a positive value, meaning the weights dictate the sign and magnitude of the controller output values. A negative controller output drives cells toward one direction, and a positive value drives cells in the opposing direction. Examples of each are shown later in the in silico section.

Using the same steps as in Equations (8) and (9), the discretized update law for the weights are as follows:

(15) $$\begin{array}{c} W_{t+1}=\left\{\begin{array}{c} W_{t}+\gamma_{a}\Phi \left(x\right)e\\ -\mathrm{sign}\left(e\right)\alpha_{a}\frac{WW^{T}\Phi \left(x\right)}{∥W∥}e,\;\;\mathrm{if}\;u>UL\\ W_{t}+\gamma_{b}\Phi \left(x\right)e\\ +\mathrm{sign}\left(e\right)\alpha_{b}\frac{WW^{T}\Phi \left(x\right)}{∥W∥}e,\;\;\mathrm{if}\;u<LL\\ \;W_{t}+\gamma \Phi \left(x\right)e,\;\;\mathrm{otherwise}. \end{array}\right. \end{array}$$

### 3.3. In Silico Feedback Control Experiments

For the in silico analysis, we include three examples. Each example consists of 100 simulations at each step (i.e., 100 cells), and the results are averaged for the model output. Moreover, we consider operational limits with EF strength bounds ranging from [−4,4] V/cm. In Example 1, the reference trajectory for directedness is limited to the range [−0.65,0.65] to prevent saturation and to demonstrate that both the new and baseline controllers exhibit similar behavior. In contrast, Examples 2 and 3 have reference trajectories for directedness bounded between [−0.9,0.9] to ensure that the control inputs exceed the actuation limits. These two examples specifically illustrate how the revised controller can mitigate saturation effects and keep control inputs within the operational limits of the devices. All design parameters of the RBF-NN used in Examples 1, 2, and 3 are detailed in Table 1.

#### 3.3.1. Example 1

**Figure 7:**
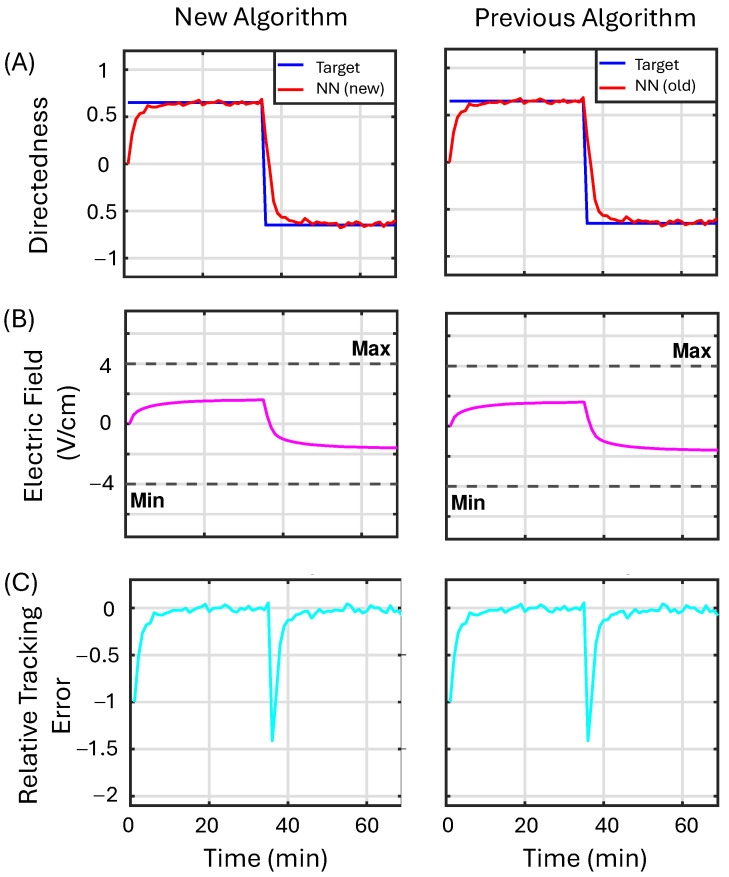
The performance of the machine learning algorithm utilizing projection (first column) is assessed against that of the original machine learning algorithm (second column) in Example 1 (see Section 3.3.1). Row (**A**) displays the model’s output in red alongside the desired reference value in blue. Row (**B**) provides a comparison between the saturated control output and the actual output generated by the controller, with the control output being saturated whenever it exceeds the dashed black lines; the actual control output is shown in magenta. Row (**C**) illustrates the relative tracking error in cyan.

This example demonstrates that for target values that do not require the control input to reach maximum EF values, both controllers behave identically. Figure 7 presents the simulation results comparing the proposed NN algorithm that includes projection on the weight updates (first column) with the standard weight update law (second column). Row (A) displays the model’s output in red alongside the desired reference value in blue. Row (B) provides a comparison between the saturated control output and the actual output generated by the controller, with the control output being saturated whenever it exceeds the dashed black lines; the actual control output is shown in magenta. Row (C) illustrates the relative tracking error in cyan. Figure 7 demonstrates that both algorithms produce similar outputs within the specified bounds and effectively maintain the target directedness (from which we can calculate the RI value) for the population of cells.

#### 3.3.2. Example 2

In Example 2, we demonstrate the revised NN controller’s ability to regulate the control input near the boundaries. To demonstrate this, we select target values for directedness with sufficiently large magnitudes that would normally result in the controller pushing to increase the EF strength beyond the allowed limits. The reference values are set to 0.9 and −0.9, for the first and second half of the simulation, respectively.

**Figure 8:**
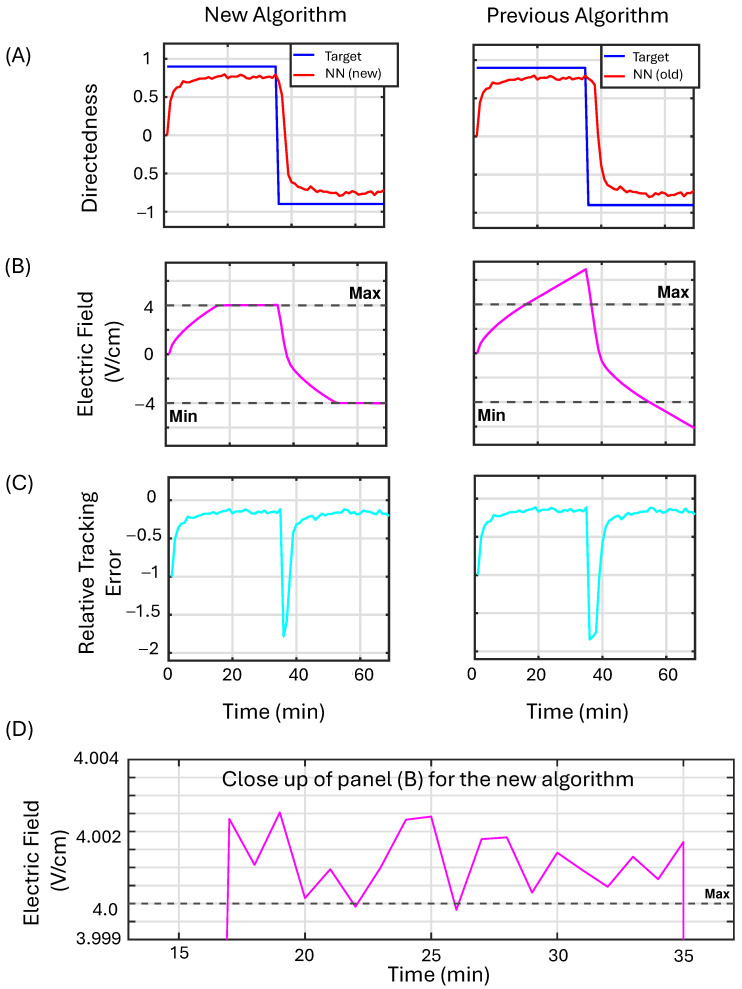
The performance of the NN controller with projection (first column) is compared to the original NN controller (second column) in Example 2 (see Section 3.3.2). In row (**A**), the model’s output is displayed in red alongside the desired reference value shown in blue. Row (**B**) compares the saturated control output with the actual output generated by the controller; the control output is considered saturated whenever it exceeds the dashed black lines, while the actual control output is represented in magenta. Row (**C**) illustrates the relative tracking error in cyan. The plot in panel (**D**) provides a closer view of the left plot in row (**B**) for the proposed algorithm with projection. We note that the controller successfully keeps the control law near the boundaries.

Figure 8 presents the simulation results that compare the proposed NN-based algorithm, which incorporates projections on the weight updates (first column), with the original algorithm (second column). In row (A), the model’s output is displayed in red alongside the desired reference value shown in blue. Row (B) compares the saturated control output with the actual output generated by the controller; the control output is considered saturated whenever it exceeds the dashed black lines, while the actual control output is represented in magenta. Row (C) illustrates the relative tracking error in cyan. The plot in panel (D) provides a closer view of the proposed algorithm with projections in row (B), demonstrating its ability to push the control output back toward the maximum allowed EF, unlike the standard algorithm, which tends to continually increase. This continuous increase in output by the standard algorithm results in a delay when the reference value changes sign.

#### 3.3.3. Example 3

For Example 3, we increase the values of $\gamma_{a,b}$, which increases the buffer region before the projection operator begins to dominate the dynamics of the weight updates. In this case, we should see a less aggressive approach to maintain the applied EF strength near the boundaries. Instead, we observe a slowed growth of the control input as the controller pushes to reach an unattainable target value.

**Figure 9:**
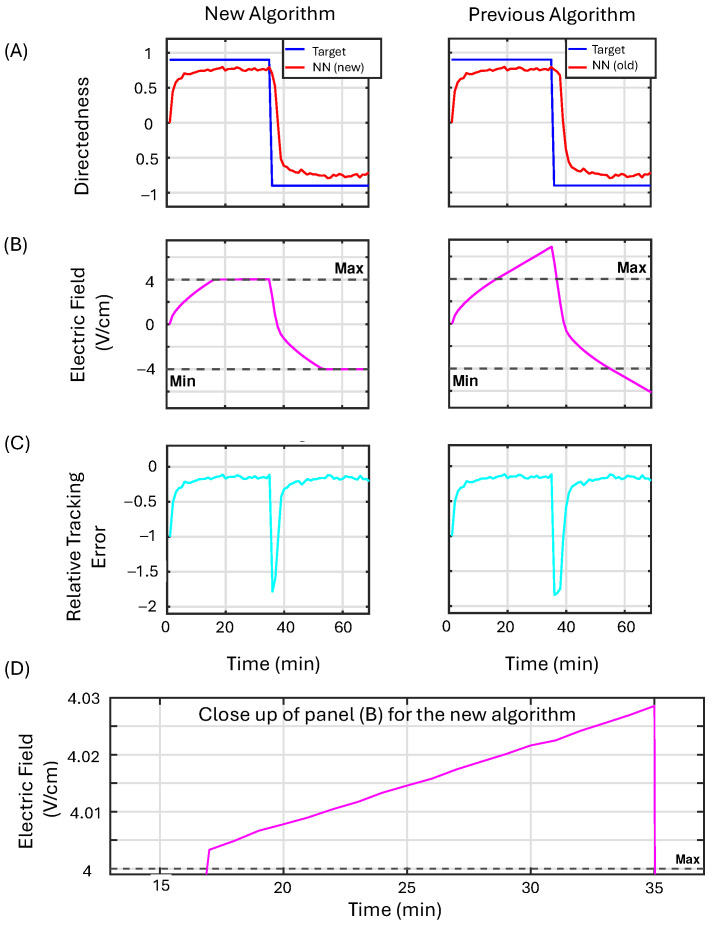
The performance of the machine learning algorithm utilizing projection (first column) is assessed against that of the original machine learning algorithm (second column) in Example 3 (see Section 3.3.3). In row (**A**), the model’s output is displayed in red alongside the desired reference value shown in blue. Row (**B**) compares the saturated control output with the actual output generated by the controller; the control output is considered saturated whenever it exceeds the dashed black lines, while the actual control output is represented in magenta. Row (**C**) illustrates the relative tracking error in cyan. The plot in panel (**D**) provides a closer view of the left plot in row (**B**).

Figure 9 presents the simulation results that compare the proposed NN controller, which incorporates projections on the weight updates (first column), with the original algorithm (second column). In row (A), the model’s output is displayed in red alongside the desired reference value shown in blue. Row (B) compares the saturated control output with the actual output generated by the controller; the control output is considered saturated whenever it exceeds the dashed black lines, while the actual control output is represented in magenta. Row (C) illustrates the relative tracking error in cyan. The plot in panel (D) provides a closer view of the proposed algorithm with projections, demonstrating its ability to slow down the growth of the control output after exceeding the bounds. This results in a decreased delay when the sign of the target directedness changes.

#### 3.3.4. Summary

The outcomes of the three examples are outlined in Table 2 and Table 3, utilizing various error metrics such as Mean Squared Error (MSE), Root Mean Squared Error (RMSE), and Mean Absolute Percentage Error (MAPE). This statistical evaluation illustrates the improved performance of the proposed NN-based algorithm. Importantly, this improved performance is marked by a notable decrease in tracking errors. This further highlights the effectiveness and performance of the proposed NN controller, showcasing tangible improvements in system responsiveness and adaptability.

Furthermore, Table 4 and Table 5 present analysis concerning Fall Time. Fall time refers to the duration required for the pulse to transition from its peak value to its lowest value.

### 3.4. In Vitro Feedback Control Experiments

To demonstrate the controller’s practical applicability, we implement it in an experiment to control the migration of naïve macrophages. This example presents the experimental result of applying feedback control to the recruitment index of naive macrophages using the proposed NN controller. We compare the results to a second experiment applying a PID controller. We note that in this experimental setup, we cannot directly control EF, but rather we control the applied current. The electric field (EF) is related to other variables such as current density (J) and current (*i*) through the basic principles of electromagnetism. Understanding the relationship among these factors is crucial for the effective design and application of stimulation devices. For more information about these relationships, see Appendix B. The initial objective is to achieve a recruitment index exceeding a reference value of RI=60%. Once this target is met, the reference value is changed to RI=−60%. All the design parameters of the NN controller used in the in vitro experiment are detailed in Table 6.

For the closed-loop experiment with the NN controller, there were 1254 cells tracked and 921 cells used to compute the RI after filtering out immobile cells (>25th percentile). Similarly, for the closed-loop experiment with the PID controller, there were 1174 cells tracked and 829 cells used to compute the RI. There was one experimental run for each, the ML and PID controllers, for a total of two experiments. Directedness is computed for each cell, and then based on that, a single RI value is computed at each time step as described in the methods. Using the RI value provides a way of controlling the collective behavior of a large population of cells despite cell-to-cell variability.

**Figure 10:**
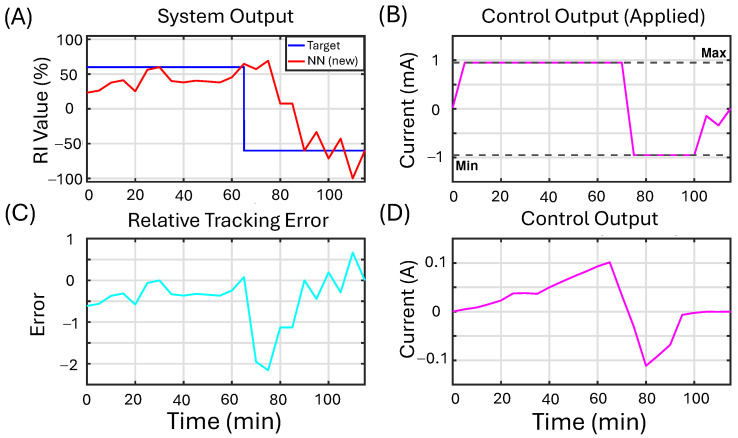
The in vitro results for the proposed NN controller are presented as follows: (**A**) This panel displays the experimental results for feedback control on the recruitment index of macrophages using the proposed NN controller. Initially, a positive reference value for the recruitment index (RI=60%), shown in blue, is set. Once this value is exceeded, the reference changes to RI=−60% for the remainder of the experiment. The red line represents the measured recruitment index value of the cells during the experimental run. (**B**) The controller output, illustrated in magenta, is clipped as indicated by the dashed black line, and the saturated control signal is transmitted to the device. (**C**) This panel shows the relative tracking error in cyan. (**D**) Here, the controller output, also depicted in magenta, represents the unsaturated control signal generated by the algorithm.

Figure 10 presents the experimental results of feedback control on the recruitment index of naive macrophages using the proposed NN controller. In the plot labeled “System Output” (Figure 10A), the blue line represents the reference value, while the red line shows the measured recruitment index of the cells throughout the experimental run. The plot titled “Control Output (Saturated/Applied)” (Figure 10B) displays the clipped control output applied to the cells. The plot titled “Control Output (Unsaturated)” (Figure 10D) illustrates the control signal generated by the controller before any saturation limits are applied. Finally, the plot titled “Relative Tracking Error” (Figure 10C) depicts the tracking error, represented in cyan.

**Figure 11:**
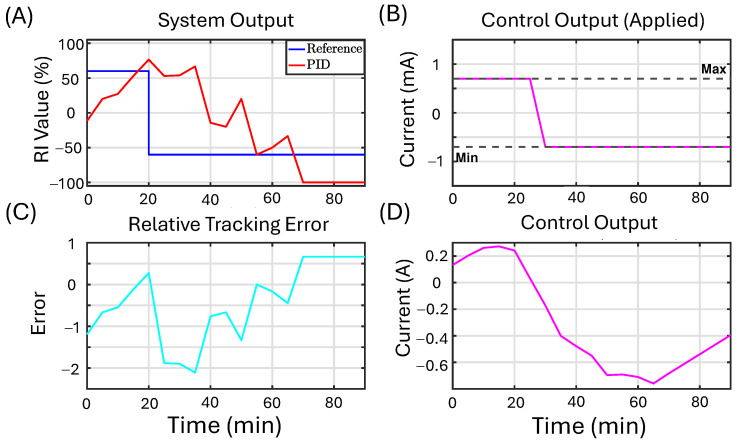
The in vitro results for the PID controller are presented as follows: (**A**) This panel displays the experimental results for feedback control on the recruitment index of macrophages using the PID controller. Initially, a positive reference value of 60% recruitment index (RI), shown in blue, is set. Once this value is exceeded, the reference changes to −60% RI, with the objective of tracking this new reference value moving forward. The red line represents the measured recruitment index value of the macrophages during the experimental run. (**B**) The controller output, illustrated in magenta, is clipped as indicated by the dashed black line, and the saturated control signal is transmitted to the device. (**C**) This panel shows the relative tracking error in cyan. (**D**) Here, the PID controller output, also depicted in magenta, represents the unsaturated control signal generated by the algorithm.

Figure 11 presents the experimental results of feedback control on the recruitment index of naive macrophages using the PID controller. Similar to the experiment performed using the NN controller, the initial objective was to achieve a recruitment index exceeding the reference value of RI=60%. Once this target was met, the reference value was changed to RI=−60% for the remainder of the experiment. In the plot labeled “System Output” (Figure 11A), the blue line represents the reference value, while the red line shows the measured recruitment index of the cells throughout the experimental run. The plot titled “Control Output (Saturated/Applied)” (Figure 11B) displays the clipped control output applied to the cells. The “Control Output (Unsaturated)” (Figure 11D) illustrates the control signal generated by the PID controller before any saturation limits were applied. Finally, the plot titled “Relative Tracking Error” (Figure 11C) depicts the tracking error, represented in cyan. We note that the PID is not able to successfully track the target value in the second part of the experiment.

One of the challenges of experiments is that due to time and cost, there are limited opportunities to tune parameters, and the algorithm should work out of the box. We note that the value chosen for $\gamma_{a}$ is not optimal. Nonetheless, the algorithm successfully reduced the control effort when compared to the second experiment, implementing a PID controller. Additionally, in accordance with our in silico study, once the initial goal was achieved, the controller rapidly adjusted its output to track the new reference value successfully.

Table 7 presents the quantitative analysis for the in vitro experiments comparing the NN and PID controllers. It is worth noting that here we are utilizing the normalized version of the error metrics used in the analysis of the in silico results.

## 4. Discussion

One of the limitations in applying the new NN controller presented stems from the uncertainty regarding the “to be” learned norm of the weights before conducting the experiments. Due to the limited number of experimental runs, there was insufficient data to identify an optimal set of parameters for the controller. As a result, the controller did not always remain close to the stability bounds. However, further experiments and improvements to the update law could help address this issue.

As previously mentioned, prolonged exposure to a strong electric field can cause polarization of macrophages [25,26,27], which may adversely affect wound healing time [28]. For example, a prematurely induced transition from pro-inflammatory to pro-regenerative macrophages may impair healing, while an accelerated recruitment of pro-inflammatory macrophages can accelerate healing [29].This highlights the need to couple this work with methods in optimal control, such as model predictive control (MPC), where one can minimize a cost function that penalizes both control effort and error. Previous work suggests the possibility of using label-free indicators such as morphological features and migratory patterns as a way to classify macrophage subtypes [30,31]. These label-free methods can be used in future work under this same experimental setup to detect possible phenotypic changes and inform adjustments in EF strength and application.

The relevance of this work directly applies to wound healing. Pro-inflammatory macrophages are abundant at the onset of inflammation, aiding in wound cleaning, while anti-inflammatory macrophages appear later, during the transition from inflammation to proliferation, helping with tissue regeneration [32]. Understanding this process allows for modulating an external electric field to enhance the recruitment of the right cell types at the appropriate stages, potentially speeding up the wound healing process. This would require real-time, in vivo monitoring of cell migration, which could be achieved by tracking biomarkers or chemical compounds in the wound bed as proxies for the relative abundance of pro-inflammatory and anti-inflammatory macrophages.

How electric fields influence macrophage behavior beyond the high-level concept of galvanotaxis remains largely unexplored. We have previously demonstrated that even the same macrophage, after phagocytosis of pathogenic bacteria, has its galvanotaxis direction reversed to the opposite direction, perfectly mirroring the well-known antigen behavior of antigen-presenting cells [33]. Cathodal migration likely involves PI3K signaling [34,35] and pSTAT3 pathways [36], as well as the anodal galvanotaxis myosin II contraction [37]. The directional response is consistent with known electrotaxis mechanisms, including polarization of intracellular signaling. Further development of a strategy to guide specific subtypes of macrophages will significantly enhance our ability to modulate biological function electrically. Although these pathways were not directly assessed in this study, PI3K signaling is likely a key mechanism. The technical approach developed here will be a useful tool to elucidate biological mechanisms further [34].

The methods presented here can be expanded to broader applications beyond cell migration. Self-regulation is a crucial feature for the health of many systems, such as in ecological dynamics modeled by predator–prey interactions [38]. In biology, this self-regulation often takes place through feedback control, where the system adjusts in response to changes to maintain stability. For example, during exercise, vasodilation occurs to increase blood flow and oxygen delivery to muscles [39]. In wound healing, distinct cell types are recruited at different stages to aid in the healing process [40]. The application of control in biological systems has already shown promise in precision medicine [41,42]. However, challenges remain in extending this work, particularly in sensing system responses in vivo.

## 5. Conclusions

The NN controller incorporating a projection operator on the weights demonstrated notable improvements over the original controller in simulations, where it was applied to a model of cell directedness. These simulations illustrated the controller’s ability to address key challenges inherent in biological systems, such as saturation effects and nonlinearities in cellular responses, which are particularly common in bioelectronic control systems. By constraining the weight updates, the projection mechanism helped ensure that the control signals remained within feasible actuation limits, preventing the undesirable saturation of the control inputs. Subsequent in vitro experiments, where the controller was used to direct the migration of naive macrophages, further validated the efficacy of this approach. The controller successfully regulated the collective migration pattern macrophages, confirming that the modification was not only theoretically sound but also practically applicable in a biological setting. This demonstrates the potential of this modified NN controller for controlling cell migration, which has significant implications for therapeutic strategies in tissue regeneration, immune response modulation, and wound healing.

The results of this study suggest that the controller is particularly well-suited for applications where the norm of the weight vector can be predetermined, or where sufficient experimental data can be gathered to approximate this norm. This is important for situations where the control signal must dynamically change between positive and negative values in response to shifting environmental or biological cues. Moreover, the controller’s robustness to uncertainties and its ability to work within practical limits make it a promising tool for more complex, real-world scenarios where biological systems are often subject to significant variability and constraints. The integration of this controller with real-time sensing and adaptive control mechanisms could further enhance its capability to manage cell migration in unpredictable or evolving environments, opening up new possibilities for precision medicine and regenerative therapies.

Future work will focus on refining the controller’s performance in a wider range of biological models, including more complex systems like multicellular interactions and tissue-level migration. We note that this study was limited to macrophages in 2D culture under a unidirectional EF. While this study used naïve macrophages, the system can also be applied to other cell types such as keratinocyte [43] and fibroblast [44], which respond to similar electric field strengths. We note that although the model was fine-tuned using in vitro data, no data was used to inform the parameters of the NN controller. Thus, it is agnostic with respect to the specific cell type. Simple tuning of the adaptation rates on the fly can adjust the controller to work for cells with varying speeds and response times if needed. Finally, as described in above, selective control of subtypes of macrophages will offer more differential control over inflammatory and reparative processes in wound healing or other disease conditions.

## Figures and Tables

**Table 1:** Detailed design parameters of the RBF-NN used in all in silico examples. In all cases, the state feeding into the NN is $z\left(n\right)={\left[r\left(n\right),y\left(n-1\right),y\left(n-2\right),y\left(n-3\right),y\left(n-4\right),y\left(n-5\right)\right]}^{T}$ and initial conditions $c_{i}\left(0\right)=l\left(i\right)*\mathrm{ones}\left(N,1\right)\in R^{N}$, $l=repmat\left(\left[0:0.01:2\right],6\right)\in R^{M}$ and $w_{i}\left(0\right)=0.0001*rand\left(\right)$.

	Example 1	Example 2	Example 3
Sampling Time ($T_{s}$)	1[s]	1[s]	1[s]
γ	0.0005	0.0005	0.0005
β	1	1	1
Number of Neurons (*M*)	201	201	201
Number of Inputs (*N*)	6	6	6
$\gamma_{a}$	0.0015	0.0015	0.075
$\gamma_{b}$	0.006	0.006	0.27
$\alpha_{a}$	0.1	0.1	0.1
$\alpha_{b}$	0.4	0.4	0.4
UL	+4	+4	+4
LL	−4	−4	−4

**Table 2:** Error metrics for new and old NN controllers. Bold values indicate metrics where the proposed method outperforms the prior method.

	Example 1			Example 2			Example 3		
Controller	MSE	RMSE	MAPE	MSE	RMSE	MAPE	MSE	RMSE	MAPE
${\mathrm{NN}}_{Old}$	0.0339	0.1841	10.7568	0.1861	0.4314	29.4855	0.1861	0.4314	29.4855
${\mathrm{NN}}_{New}$	0.0339	0.1841	10.7568	**0.1230**	**0.3507**	**25.5900**	**0.1265**	**0.3556**	**25.8044**

**Table 3:** Percentage increase/decrease in MSE, RMSE, and MAPE using new NN controller. Bold values indicate metrics where the proposed method outperforms the prior method.

Example 1 Improve (%)			Example 2 Improve (%)			Example 3 Improve (%)		
MSE	RMSE	MAPE	MSE	RMSE	MAPE	MSE	RMSE	MAPE
0	0	0	**33.9214**	**18.7113**	**13.2115**	**32.0555**	**17.5716**	**12.4847**

**Table 4:** Fall time for new and old NN controllers. Bold values indicate metrics where the proposed method outperforms the prior method.

	Example 1	Example 2	Example 3
Controller	Fall Time (min)	Fall Time (min)	Fall Time (min)
${\mathrm{NN}}_{Old}$	2.8366	10.3362	10.3362
${\mathrm{NN}}_{New}$	2.8366	**8.0239**	**8.3560**

**Table 5:** Increase/decrease in fall time using new NN controller. Bold values indicate metrics where the proposed method outperforms the prior method.

Example 1		Example 2		Example 3	
Improve (%)	Improve (s)	Improve (%)	Improve (min)	Improve (%)	Improve (min)
0	0	**22.3706**	**2.3123**	**19.1583**	**1.9802**

**Table 6:** Detailed design parameters of the RBF-NN used in the in vitro experiment.

	In Vitro Experiment
Sampling Time ($T_{s}$)	300[s]
γ	0.00009
β	1
Number of Neurons (*M*)	101
Number of Inputs (*N*)	6
$w_{i}\left(0\right)$	0.00001∗rand()
$c_{i}\left(0\right)$	$l\left(i\right)*\mathrm{ones}\left(N,1\right)\in R^{N}$, $l=repmat\left(\left[-5:0.1:5\right],6\right)\in R^{M}$
z(n) for ML Controller	$\frac{1}{50}\times {\left[r\left(n\right),y\left(n-1\right),r\left(n-1\right),y\left(n-2\right),r\left(n-2\right),y\left(n-3\right)\right]}^{T}$
$\gamma_{a}$	0.00009
$\gamma_{b}$	0.00009
$\alpha_{a}$	0.4
$\alpha_{b}$	0.4
UL	+0.00095
LL	−0.00095

**Table 7:** Quantitative analysis for in vitro experiments. Bold values indicate metrics where the proposed method outperforms the prior method.

Controller	nMSE	nMSE Improve (%)	nRMSE	nRMSE Improve (%)	nMAPE	nMAPE Improve (%)	Fall Time (min)	Fall Time Improve (%)	Fall Time Improve (min)
PID	1.0054	–	1.0027	–	81.0202	–	18.0971	–	–
${\mathrm{NN}}_{New}$	**0.5703**	**43.2736**	**0.7552**	**24.6831**	**52.1033**	**35.6910**	**12.3886**	**31.5434**	**5.7084**

**Figure A1:**
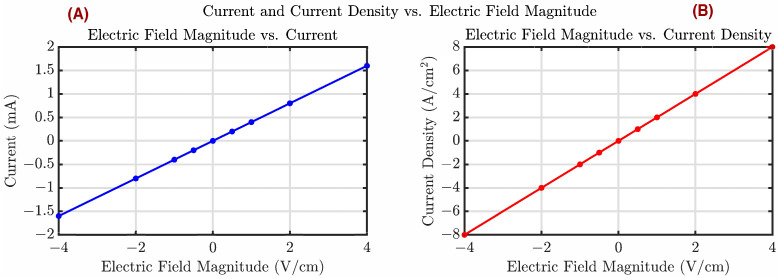
Electric field, current density, and current relations. (**A**) shows the relation between electric field magnitude and the current value and (**B**) shows the relation between electric field magnitude and the current density.

**Figure A2:**
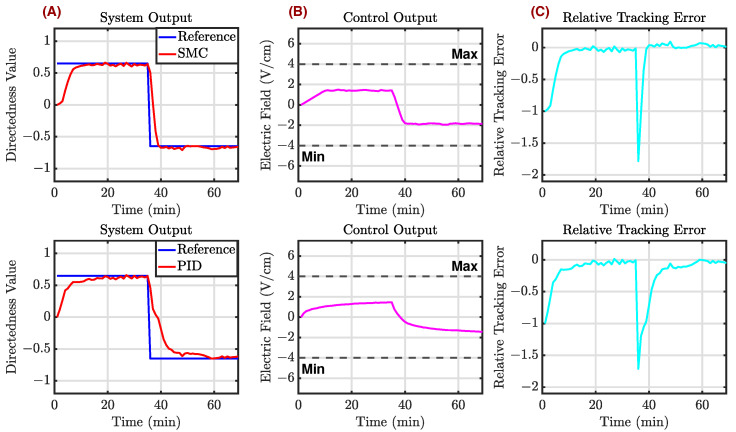
The performance of the SMC (first row) and PID (second row) in Example 1 (see Appendix C.1). Column (**A**) displays the model’s output in red alongside the desired reference value in blue. Column (**B**) provides a comparison between the saturated control output and the actual output generated by the controller, with the control output being saturated whenever it exceeds the dashed black lines; the actual control output is shown in magenta. Column (**C**) illustrates the relative tracking error in cyan.

**Figure A3:**
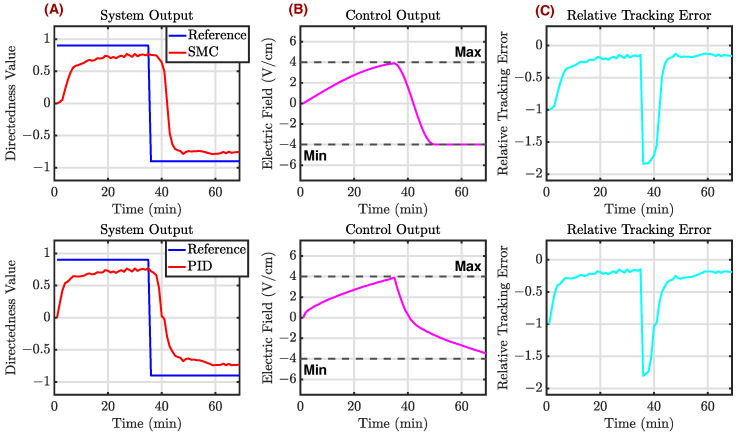
The performance of the SMC (first row) and PID (second row) in Example 2 (see Appendix C.2). Column (**A**) displays the model’s output in red alongside the desired reference value in blue. Column (**B**) provides a comparison between the saturated control output and the actual output generated by the controller, with the control output being saturated whenever it exceeds the dashed black lines; the actual control output is shown in magenta. Column (**C**) illustrates the relative tracking error in cyan.

**Figure A4:**
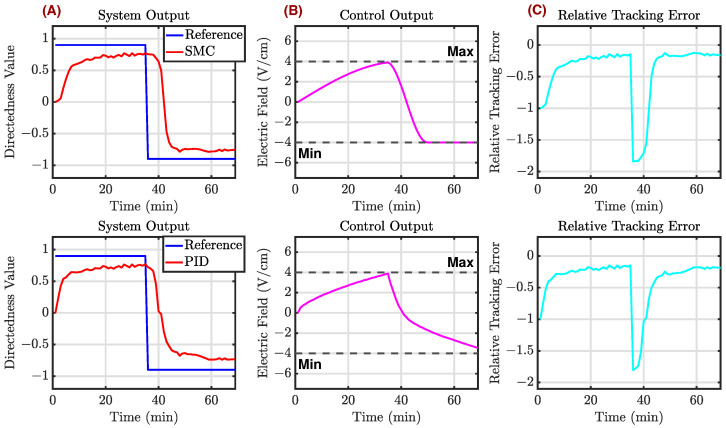
The performance of the SMC (first row) and PID (second row) in Example 3 (see Appendix C.3). Column (**A**) displays the model’s output in red alongside the desired reference value in blue. Column (**B**) provides a comparison between the saturated control output and the actual output generated by the controller, with the control output being saturated whenever it exceeds the dashed black lines; the actual control output is shown in magenta. Column (**C**) illustrates the relative tracking error in cyan.

## Data Availability

All the data/code is available through Github Controlling-Cell-Migratory-bioengineering, (accessed on 10 June 2025).

## Appendix A. Lyapunov Analysis

To follow the stability analysis as in [45], we say the error dynamics are as follows:

(A1) $$\begin{array}{c} \left\{\begin{array}{cc} \dot{e} & =-e+\left(W^{*}-W\right)\Phi \left(t\right)\\ \dot{W} & =e\Phi (t) \end{array}\right. \end{array}$$

where Φ(t) is as in Equation (4), which is a bounded function of time t. We say that $W^{*}$ gives the optimal weight values to track the reference, which are constant. To show that the error tends to 0, e(t)→0, we define a Lyapunov candidate as

(A2) $$V=e^{2}+{\left(W^{*}-W\right)}^{2}$$

Taking the derivative with respect to time, we arrive at

(A3) $$\begin{array}{cc} \dot{V} & =2e\dot{e}+2\left(W^{*}-W\right)\left(-\dot{W}\right)\\ & =2e\left(-e+\left(W^{*}-W\right)\Phi \left(t\right)\right)+2\left(W^{*}-W\right)\left(-e\Phi \left(t\right)\right)\\ & =-2ee+2e\left(W^{*}-W\right)\Phi \left(t\right)-2e\left(W^{*}-W\right)\Phi \left(t\right)\\ & =-2e^{2}\leq 0. \end{array}$$

This means V(t) is decreasing as a function of time. Thus, e(t) and W(t) are uniformly bounded. Since V(t) is decreasing and lower-bounded, it must tend towards a limit. Taking the second time derivative, we get

(A4) $$\begin{array}{cc} \ddot{V} & =-4e\dot{e}\\ & =-4e(-e+\left(W^{*}-W\right)\Phi \left(t\right)). \end{array}$$

Since Φ(t) is bounded since it is a Gaussian function, and e(t) and W(t) were shown to be bounded, this means $\ddot{V}\left(t\right)$ is uniformly bounded and that V(t) is uniformly continuous. Thus, by Barbalat’s lemma, $\dot{V}\left(t\right)\to 0$ as t→∞. This means the error tends towards zero as t→∞.

## Appendix B. Electric Field, Current Density, and Current Relations

Electric fields (EF), current density (J), and current (*i*) are fundamental parameters in the context of electrical stimulation for chronic wound healing and pain relief. These variables are intricately linked through fundamental principles of electromagnetism, and understanding their relationship is essential for the proper design and application of stimulation devices.

In clinical studies involving electrical stimulation, the electric field (EF) is typically applied via devices delivering direct current (DC), or monophasic or biphasic pulsed current. The voltage range for these devices typically spans from 20 to 500 V, and the current used ranges from 200 to 800 mA [46]. These applied electric fields can stimulate biological processes, particularly in the context of wound healing and pain management. Many biological cells are sensitive to the electric fields applied during such treatments. These cells, including keratinocytes, fibroblasts, and neurons, exhibit behaviors such as migration in response to electric fields—a phenomenon known as electrotaxis or galvanotaxis. In particular, most cells tend to migrate toward the cathode (negative electrode) under the influence of an electric field. This migration is optimal in fields on the order of 100 mV/mm. In the context of wound healing, the electric fields generated in the body are typically in the range of 400 mV/mm (up to 4 V/cm), as reported in studies involving human and mouse skin wounds [47,48]. Such fields are believed to promote cellular migration and tissue regeneration, helping to accelerate healing.

Current density (J) is defined as the amount of electric current flowing per unit area. It is directly related to the electric field (EF) through the material’s electrical conductivity (σ), as given by Ohm’s law [49]:

(A5) J=σ·EF

Here, J is the current density (measured in amperes per square meter, A/m^2^). σ is the electrical conductivity of the medium (measured in Siemens per meter, S/m). EF is the electric field (measured in volts per meter, V/m). Electrical conductivity (σ) is the inverse of resistivity (ρ), meaning that materials with high conductivity allow current to flow easily, while materials with high resistivity impede current flow. In clinical settings, the medium through which the electric field is applied (e.g., tissue, conductive gel, or saline solution) significantly influences the current density and, consequently, the effectiveness of the treatment.

In many studies, including those involving electrotaxis, the current density is calculated using the known conductivity of the medium and the electric field applied. For example, if the resistance is known, the voltage can be determined, and vice versa, provided the relationship between current, voltage, and resistance is understood. For instance, in our study setup, with a known resistance of 50Ω-cm and an electric field ranging from −4 to 4 V/cm, we can calculate the current density. If the current density (J) is known and the area through which the current passes is specified (e.g., $0.02\;{\mathrm{cm}}^{2}$), the electric field can be determined using Equation (A5). Figure A1 shows the electric fields (EF), current density (J), and current (*i*) relations.

This relationship ensures that the current applied during the experiment remains within the desired parameters, ensuring consistent voltage and current throughout the experiment. Monitoring instruments such as voltmeters and digital multimeters are typically used to verify these conditions in real time, confirming that the voltage and current remain stable throughout the experiment, particularly for longer durations.

## Appendix C. Additional In Silico Comparisons with Existing Controllers

For the in silico analysis, we included three additional comparisons with standard methods, such as proportional–integral–derivative (PID) [50,51] controller and sliding-mode controller (SMC) [52,53,54]. In all simulations, the parameter *s* is set to 3, which makes it easier for the cells to move toward the cathode with less electric field (EF) required. The delay parameter τ is set to 4 to align with the transient behavior observed in the macrophage data. Each example consists of 100 simulations at each step, and the results are averaged for the model output. Moreover, we consider the operational limits of the devices, with EF strength bounds ranging from [−4,4] V/cm. In Example 1, the trajectory for directedness is limited to the range [−0.65,0.65] to prevent saturation and to demonstrate that both the new and old controllers exhibit similar behavior. In contrast, Examples 2 and 3 have the directedness trajectory bounded between [−0.9,0.9] to ensure that the control input exceeds the actuation limits. These two examples specifically illustrate how the revised controller can mitigate saturation effects and keep control inputs within the operational limits of the devices. All design parameters of the RBF-NN used in Examples 1, 2, and 3 are detailed in Table 1.

It is important to point out that to ensure an unbiased evaluation, each controller is calibrated only one time, as the objective is to evaluate the performance of all controllers while keeping their initial configurations. Essentially, there are no additional modifications made to the controllers to adapt to the altered system conditions.

### Appendix C.1. Example 1

Figure A2 presents the simulation results of SMC (top row) and PID (second row). Column (A) displays the model’s output in red alongside the desired reference value in blue. Column (B) provides a comparison between the saturated control output and the actual output generated by the controller, with the control output being saturated whenever it exceeds the dashed black lines; the actual control output is shown in magenta. Column (C) illustrates the relative tracking error in cyan. Figure A2 demonstrates that both controllers effectively maintain the directedness of the cells for successful trajectory tracking.

### Appendix C.2. Example 2

Figure A3 presents the simulation results of SMC (top row) and PID (second row). Column (A) displays the model’s output in red alongside the desired reference value in blue. Column (B) provides a comparison between the saturated control output and the actual output generated by the controller, with the control output being saturated whenever it exceeds the dashed black lines; the actual control output is shown in magenta. Column (C) illustrates the relative tracking error in cyan. Figure A3 demonstrates that both controllers effectively maintain the directedness of the cells for successful trajectory tracking.

### Appendix C.3. Example 3

Figure A4 presents the simulation results of SMC (top row) and PID (second row). Column (A) displays the model’s output in red alongside the desired reference value in blue. Column (B) provides a comparison between the saturated control output and the actual output generated by the controller, with the control output being saturated whenever it exceeds the dashed black lines; the actual control output is shown in magenta. Column (C) illustrates the relative tracking error in cyan. Figure A4 demonstrates that both controllers effectively maintain the directedness of the cells for successful trajectory tracking.

The outcomes of all these examples are outlined in Table A1, utilizing various error metrics such as Mean Squared Error (MSE), Root Mean Squared Error (RMSE), and Mean Absolute Percentage Error (MAPE). This statistical evaluation illustrates the improved performance of the proposed NN-based methodology. Importantly, this improved performance is marked by a notable decrease in tracking errors. This further highlights the effectiveness and performance of the ML-based approach, showcasing tangible improvements in system responsiveness and adaptability.

**Table A1:** Error metrics for new ML, SMC, and PID controllers. Old values indicate metrics where the proposed method outperforms the prior method.

	Example 1			Example 2			Example 3		
Controller	MSE	RMSE	MAPE	MSE	RMSE	MAPE	MSE	RMSE	MAPE
${\mathrm{NN}}_{New}$	**0.0339**	**0.1841**	**10.7568**	**0.1230**	**0.3507**	**25.5900**	**0.1265**	**0.3556**	**25.8044**
SMC	0.0540	0.2324	14.4509	0.3161	0.5622	40.3215	0.3161	0.5622	40.3215
PID	0.0657	0.2563	20.6002	0.2397	0.4895	37.9279	0.2397	0.4895	37.9279
